# Impact of contrast volume on medium- to long-term renal function in patients undergoing repeated percutaneous coronary interventions

**DOI:** 10.1016/j.ijcha.2026.101974

**Published:** 2026-09-02

**Authors:** Philipp Steinhoff, Christian M. Valina, Derek Hazard, Miroslaw Ferenc, Malte Niehoff, Dirk Westermann, Franz-Josef Neumann, Philipp Breitbart, Philipp Ruile

**Affiliations:** a Department of Cardiology and Angiology, Medical Center-University of Freiburg, Faculty of Medicine, University of Freiburg, Südring 15, 79189 Bad Krozingen, Germany; b Institute of Medical Biometry and Statistics, Faculty of Medicine and Medical Center, University of Freiburg, Freiburg, Germany; c Cardioangiological Center Bethanien (CCB), Frankfurt, Germany; Im Prüfling 23, 60389 Frankfurt, Germany

**Keywords:** Percutaneous coronary intervention, Renal function, Contrast-associated kidney injury, Complication prevention, Interventional cardiology

## Abstract

**Background:**

Contrast-associated nephropathy is a complication of cardiovascular procedures. Although contrast volume is a risk factor for acute kidney injury, its impact on medium- to long-term renal function remains unknown. This study aimed to investigate this association in patients undergoing repeated percutaneous coronary interventions (PCI).

**Methods:**

This retrospective single-centre study included patients undergoing repeated PCIs between 2003 and 2023, with intervals of ≥7 and ≤ 365 days between procedures. Renal function was classified by “Kidney Disease: Improving Global Outcomes” (KDIGO) staging system. The maximum allowable contrast dose (MACD) was retrospectively calculated as individual threshold. Primary endpoint was progression to a higher KDIGO stage at readmission. Secondary endpoints included changes in estimated glomerular filtration rate (eGFR).

**Results:**

In total 6714 patients were included (mean age 67.2 years; 80.2% male; KDIGO stage analysis available for 6611; median interval between procedures 127 days). Baseline mean eGFR was 74.4 mL/min/1.73 m^2^. Median contrast volume was 220 mL, and median MACD 300 mL. At readmission, mean eGFR was 72.2 mL/min/1.73 m^2^, 1700 Patients (25.7%) showed KDIGO stage progression. Neither contrast volume nor MACD exceedance was associated with renal deterioration. However, predispositions (diabetes mellitus, arterial hypertension, age), clinical presentation (STEMI, NSTEMI) and the vascular access route were significantly associated with adverse renal outcomes.

**Conclusions:**

Contrast volume and MACD exceedance during PCI was not associated with deterioration of medium- to long-term renal function.

**Abbreviations:**

**Table:** 

PCI:	Percutaneous coronary intervention
eGFR:	Estimated glomerular filtration rate
KDIGO:	Kidney Disease: Improving Global Outcomes
MACD:	Maximum allowable contrast dose
CA-AKI:	Contrast-associated acute kidney injury
BMI:	Body mass index
HR:	Hazard ratio
CI:	Confidence interval
NSTEMI:	Non-ST-segment elevation myocardial infarction
STEMI:	ST-segment elevation myocardial infarction

## Graphical abstract

**Figure:**
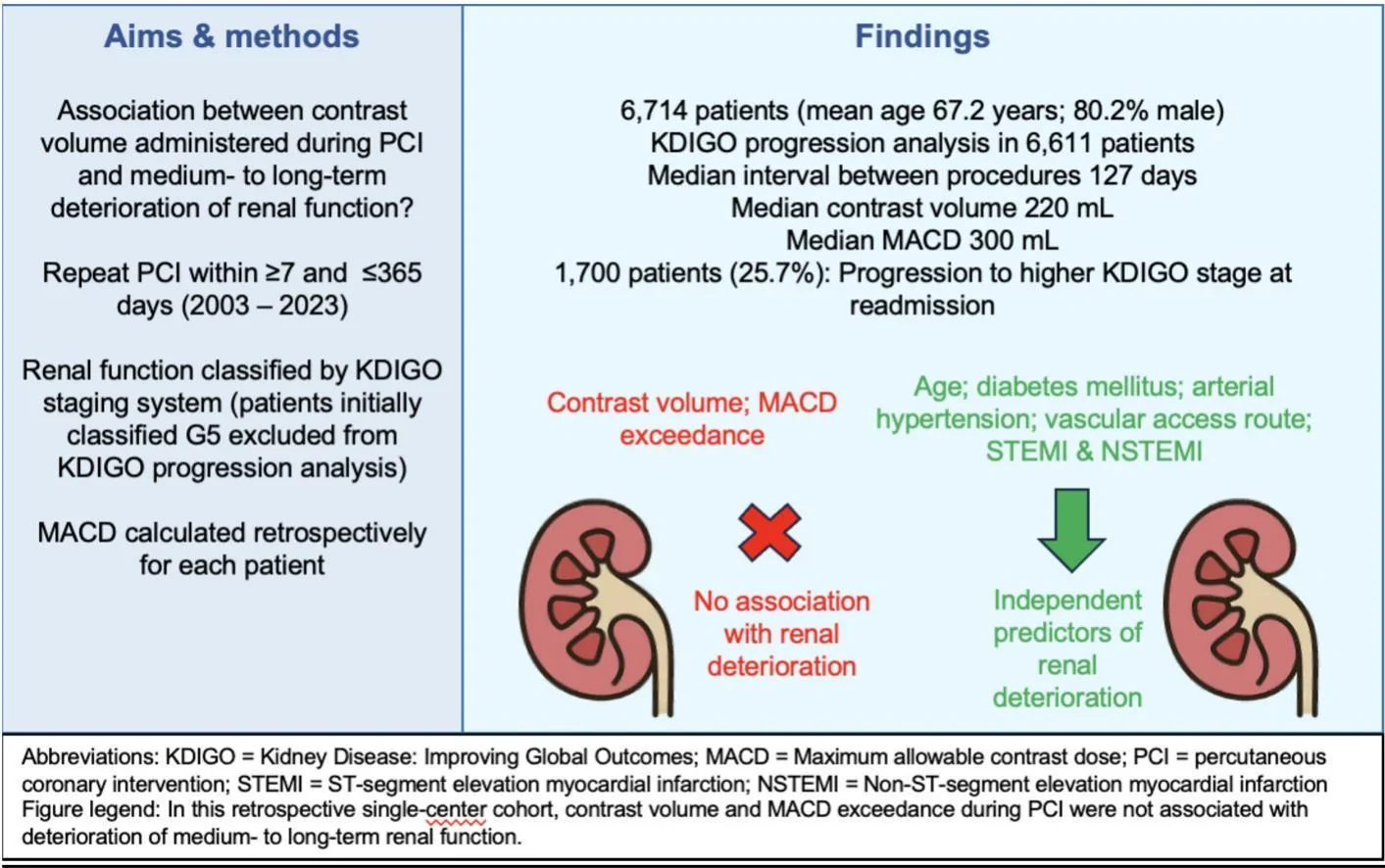

## 1 Introduction

Contrast-associated acute kidney injury (CA-AKI) is a significant complication associated with the use of iodine-containing contrast agents in cardiovascular procedures and is linked to adverse clinical outcomes, including increased in-hospital mortality, prolonged hospital stays, and higher healthcare costs [1], [2], [3], [4]. CA-AKI occurs in approximately 7% of patients undergoing percutaneous coronary intervention (PCI) [4].

Several modifiable and non-modifiable risk factors for CA-AKI have been identified [5]. Modifiable factors include extracellular volume depletion [5], vascular access route [1], [6], [7], [8], [9], as well as the type and – most notably - the volume of contrast agent administered [1], [5].

For decades, higher volumes of iodine-containing contrast agents have been considered acutely nephrotoxic [1], [5]. Earlier studies proposed fixed volume cut-offs as thresholds for CA-AKI [10], [11], whereas more recent approaches advocate for individualized risk assessment [1]. The maximum allowable contrast dose (MACD), which incorporates body weight and baseline renal function (via serum creatinine), provides a patient-specific contrast threshold [12].

In contrast, recent data on intravenous contrast administration suggest that CA-AKI risk may be driven more by underlying comorbidities than by the contrast agent itself. However, these findings cannot be extrapolated to cardiovascular interventions [13].

Existing evidence on the impact of contrast volume administered during cardiovascular procedures focuses primarily on short-term renal effects. While acute kidney injury following coronary angiography has been linked to long-term renal decline [14], direct evidence relating the contrast volume used during PCI to medium- or long-term kidney function is lacking. Given the substantial clinical and economic impact of kidney disease [15] – and the fact that contrast volume is a modifiable risk factor [1], [5] - clarifying this association is of crucial importance.

This study evaluates the association between contrast volume during an initial PCI and renal function at readmission in patients undergoing repeated PCI within 12 months.

## 2 Methods

### 2.1 Study population

This single-center, retrospective observational study included all patients who underwent repeated PCI for any clinical indication between 2003 and 2023, with an interval of ≥7 days and ≤ 365 days between procedures.

### 2.2 Data collection

All clinical and follow-up data were retrieved from our institutional database without additional testing.

Written informed consent for the use of anonymized clinical, procedural, and follow-up information was obtained from all patients at the time of the initial intervention. The study was approved by the institutional review board of the University of Freiburg (ethics approval reference number 23–1214-S1-retro, dated 09.07.2024) and conducted in accordance with the principles of the Declaration of Helsinki and the STROBE guidelines [16].

Data collection included baseline characteristics, admission dates, clinical presentations, cardiovascular and renal risk factors and laboratory parameters of renal function – specifically estimated glomerular filtration rate (eGFR) - at baseline (pre-procedure) and at readmission. The volume of contrast agent administered during the initial PCI and the vascular access route were also recorded.

### 2.3 Definitions

Kidney function was classified according to “Kidney Disease: Improving Global Outcomes” (KDIGO) guidelines [15]. Patients were retrospectively assigned to their respective KDIGO stage based on eGFR values at each time point to objectively assess relevant changes in kidney function. KDIGO stages included G1 (normal or high, eGFR ≥90 mL/min/1.73 m^2^), G2 (mildly decreased, eGFR 60–89 mL/min/1.73 m^2^), G3a (mild to moderately decreased, eGFR 45–59 mL/min/1.73 m^2^), G3b (moderately to severely decreased, eGFR 30–44 mL/min/1.73 m^2^), G4 (severely decreased, eGFR 15–29 mL/min/1.73 m^2^) and G5 (kidney failure, eGFR <15 mL/min/1.73 m^2^).

A decline in renal function was defined as any transition to a higher KDIGO stage, including substage progression within stage 3 (e.g., from G3a to G3b). Patients classified as G5 at baseline were therefore excluded from the analysis regarding KDIGO stage progression because further deterioration could not be captured within the KDIGO classification.

Recommended additional assessment based on albuminuria was not performed due to the lack of routine measurements during the included patients' hospital stays.

Furthermore, for each patient, the individual MACD was retrospectively calculated to analyze the effects of the actual contrast agent volume used on medium- to long-term kidney function, taking these individualized thresholds into account. MACD was calculated according to the original formula: 5 x body weight (in kg) / serum creatinine (in mg/dL), with a maximum dose of 300 mL [12]. Therefore, patients with a calculated MACD over the maximum defined value of 300 mL were assigned a MACD of 300 mL.

The primary endpoint was renal function decline at readmission, defined as progression to a higher KDIGO stage [15]. Secondary endpoints were changes in eGFR analyzed in relation to contrast volume during the index PCI. Multivariable models were constructed to identify factors associated with an increased risk of renal deterioration at readmission. Variable selection was guided by the inclusion of standard patient characteristics with broad clinical relevance (age, sex, and BMI), the inclusion of procedural variables of interest (contrast volume administered during index PCI and vascular access route), the clinical presentation at the time of the initial PCI (acute coronary syndrome, chronic coronary syndrome) and the inclusion of leading epidemiological risk factors for nephrotoxicity (arterial hypertension and diabetes mellitus) [15] as well as baseline renal function. In order to maintain conceptual homogeneity between the metric used to characterize baseline renal function and the metric used to define the outcome in each respective analysis, KDIGO stage at the time of index PCI was included as a covariate in the KDIGO-based endpoint model, and eGFR at the time of index PCI in the eGFR-based endpoint model.

### 2.4 Statistical analysis

Categorical variables were expressed as counts and percentages, and continuous variables as means ± standard deviations or median with interquartile range as appropriate. Variables with markedly skewed distributions (contrast volume) were natural-log transformed prior to analysis. Potential multicollinearity among covariates was evaluated by calculating variance inflation factors (VIFs).

We modeled time to event using an interval-censored Cox proportional hazards model (R package icenReg) because a decline in KDIGO stage could occur at a time before the follow-up PCI. Proportional hazards assumptions were checked via Schoenfeld residuals, and confidence intervals were obtained from 1000 bootstrap replicates.

For continuous outcomes at follow-up PCI (eGFR), we fitted linear regression models adjusted for the corresponding pre-PCI value. To allow for a non-linear relationship between time to second PCI and the outcome, days from first to second PCI were incorporated using natural cubic splines. Model diagnostics—including plots of residuals versus fitted values—were inspected visually to assess homoscedasticity and model fit. All regression models were fit with readmission values as the dependent variable, adjusting for baseline values to assess the change between the two time points.

A sensitivity analysis substituted total contrast volume with a binary indicator of whether the MACD was exceeded; all primary analyses were then repeated using this alternative exposure definition.

All statistical analyses were conducted in R 4.2.2 (R Foundation for Statistical Computing, Vienna, Austria). Two-sided *P*-values < 0.05 were considered statistically significant, and all estimates are presented with 95% confidence intervals. Given the negligible proportion of missing data (<0.1%), analyses were conducted using a complete case approach.

## 3 Results

### 3.1 Study population

A total of 6714 patients were included, with a mean age of 67.2 ± 10.4 years; 80.2% were male, and the mean body mass index (BMI) was 28.0 kg/m^2^. Arterial hypertension was present in 73.1% of patients, and 30.1% had diabetes mellitus.

Baseline renal function showed a mean eGFR of 74.4 mL/min/1.73 m^2^. According to KDIGO classification, 25.5% of patients were in stage G1, 51.8% in G2, 13.0% in G3a, 6.2% in G3b, 2.0% in G4, and 1.5% in G5. Since 85% of the patients (5716 patients) had a MACD of 300 mL, the median MACD was also 300 mL. The median contrast agent volume administered during PCI was 220 mL [170, 300].

The median interval between procedures was 127 days [67.0, 191.0]. In 4041 cases (60.2%), the initial PCI was performed via femoral access. In 2385 patients (35.5%), the initial PCI was performed for acute coronary syndrome. At the time of readmission, the mean eGFR was 72.2 mL/min/1.73 m^2^. At this time point, 22.1% of patients were in KDIGO stage G1, 51.9% in G2, 14.3% in G3a, 7.7% in G3b, 2.2% in G4, and 1.7% in G5. After excluding patients initially in KDIGO stage G5, an analysis regarding KDIGO stage progression at readmission could be performed in 6611 of the 6714 patients. Among these, 1700 patients (25.7%) showed progression to a higher KDIGO stage.

A detailed summary of the patient characteristics and the clinical presentations at the time of the initial PCI as well as at readmission for the repeat PCI is shown in Table 1. A graphical comparison of the KDIGO stage distribution at the respective time points is shown in Fig. 1.

**Table 1:** Patient characteristics at baseline and at readmission for repeat PCI.

Patient characteristics at baseline	All patients (*N* = 6714)
**Age (years)**	67.2 ± 10.4; missing: 0 (0.0)
**Male**	5386 (80.2); missing: 0 (0.0)
**BMI (kg/m**^**2**^**)**	28.0 ± 4.3; missing: 3 (0.0)
**Arterial hypertension**	4909 (73.1); missing: 0 (0.0)
**Diabetes mellitus**	2020 (30.1); missing: 0 (0.0)
**eGFR (mL/min/1.73 m**^**2**^**)**	74.4 ± 21.5; missing: 0 (0.0)
**KDIGO**	missing: 0 (0.0)
G1	1712 (25.5)
G2	3480 (51.8)
G3a	873 (13.0)
G3b	416 (6.2)
G4	134 (2.0)
G5	101 (1.5)
**MACD (mL)**	300.0 [300, 300]; missing: 3 (0.0)
**Access route**	missing: 173 (2.6)
Femoral	4041 (60.2)
Radial	2500 (37.2)
**Applied contrast volume (mL)**	220 [170, 300]; missing: 0 (0.0)
**Clinical presentation**	missing: 0 (0.0)
Acute coronary syndrome	2385 (35.5)
STEMI	678 (10.1)
NSTEMI	1095 (16.3)
Unstable angina	612 (9.1)
Chronic coronary syndrome	4117 (61.3)
Asymptomatic	212 (3.2)
**Patient characteristics at repeat PCI**	
**Interval between PCI procedures (days)**	127 [67, 191]; missing: 0 (0.0)
**eGFR (mL/min/1.73 m**^**2**^**)**	72.2 ± 21.6; missing: 0 (0.0)
**KDIGO**	missing: 0 (0.0)
G1	1482 (22.1)
G2	3484 (51.9)
G3a	963 (14.3)
G3b	519 (7.7)
G4	150 (2.2)
G5	116 (1.7)
**KDIGO stage progression**	1700 (25.7); missing: 0 (0.0)
**Clinical presentation**	missing: 0 (0.0)
Acute coronary syndrome	1020 (15.2)
STEMI	73 (1.1)
NSTEMI	479 (7.2)
Unstable angina	468 (7.0)
Chronic coronary syndrome	4694 (69.9)
Asymptomatic	1000 (14.9)

Values are mean ± SD or median [interquartile range] where appropriate, n (%). PCI denotes percutaneous coronary intervention; BMI denotes body mass index; eGFR denotes estimated glomerular filtration rate; KDIGO denotes “Kidney Disease: Improving Global Outcomes” classification, MACD denotes maximum allowable contrast dose, STEMI denotes ST-segment elevation myocardial infarction; NSTEMI denotes Non-ST-segment elevation myocardial infarction.

**Fig. 1:**
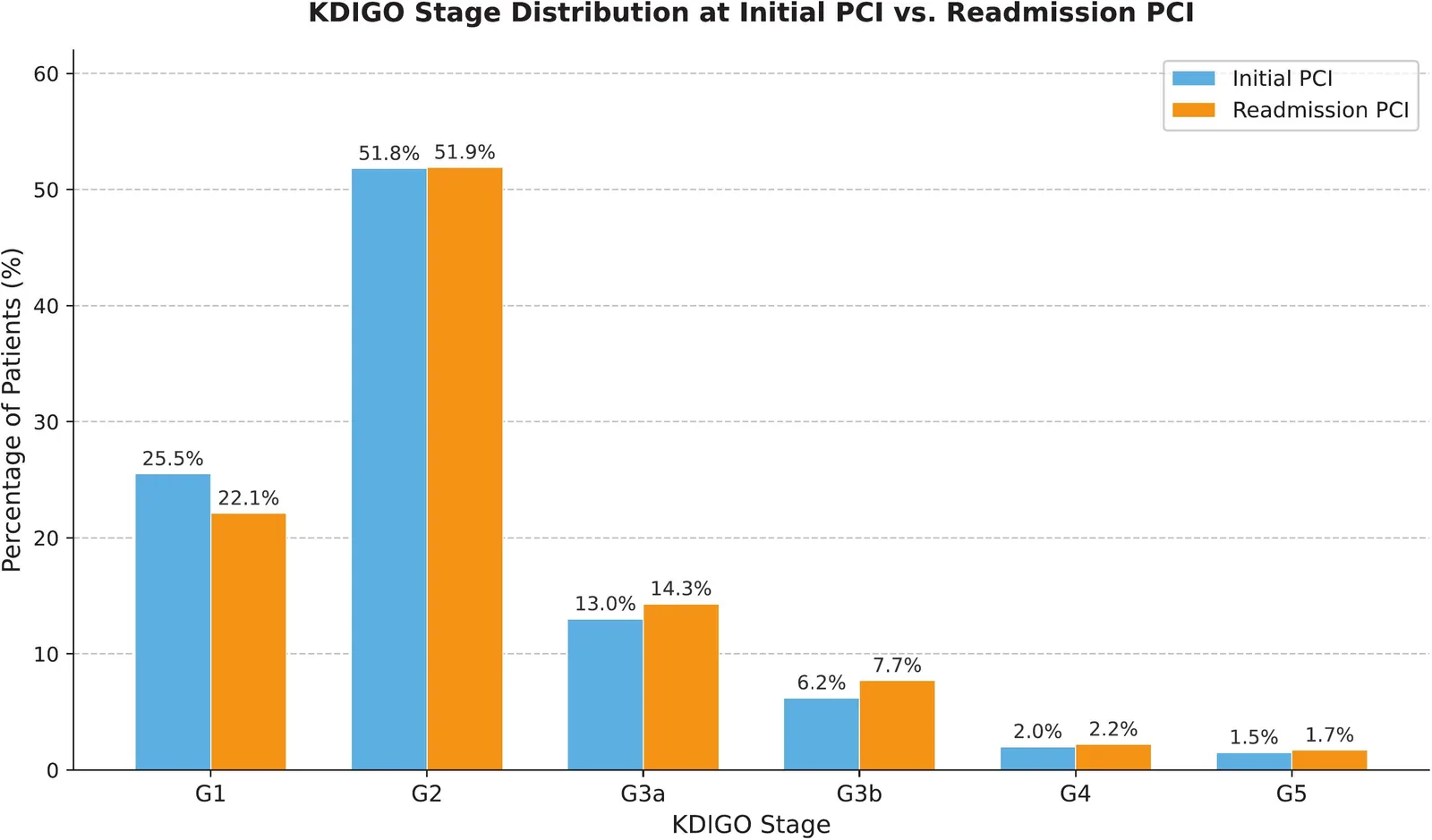
KDIGO Stage Distribution at initial PCI and at Readmission PCI. KDIGO denotes “Kidney Disease: Improving Global Outcomes” classification, PCI denotes percutaneous coronary intervention.

### 3.2 Univariable and multivariable Cox proportional hazards model analysis of potential predictors of progression to a higher KDIGO stage at readmission

In the univariable analysis, no statistically significant association was observed between the contrast volume administered during the initial PCI and progression to a higher KDIGO stage at readmission (Hazard Ratio: 1.08, 95% Confidence Interval: 0.98–1.19, *p* = 0.1248).

Similarly, in the multivariable analysis, the initial contrast volume was not significantly associated with KDIGO stage progression. Furthermore, no statistically significant associations were found for the sex or BMI. In contrast, progression to more advanced KDIGO stages at readmission was significantly associated with age, arterial hypertension, diabetes mellitus, not using a radial vascular access, as well as STEMI and NSTEMI. On the other hand, higher initial KDIGO stages were significantly associated with a lower risk of further progression to a worse stage by the time of rehospitalization. Compared to the reference group (KDIGO G1), the following hazard ratios were observed: KDIGO G2 (HR 0.23; 95% CI: 0.2–0.26), G3a (HR 0.17; 95% CI: 0.14–0.2), G3b (HR 0.07; 95% CI: 0.05–0.11), and stage 4 (HR 0.11; 95% CI: 0.08–0.19).

### 3.3 Univariable and multivariable linear regression analysis of potential predictors of eGFR at readmission

In the univariable linear regression analysis, the contrast volume administered during the initial PCI showed no statistically significant association with eGFR (Estimate: 0.31; 95% CI: −0.64-1.25) at follow-up.

In the multivariable linear regression analysis, diabetes mellitus (Estimate: -3.13; 95% CI: −4.11 – −2.14), arterial hypertension (Estimate: -2.88; 95% CI: −3.90 - -1.86), age (Estimate: -0.13; 95% CI: −0.18 - -0.08) and NSTEMI (Estimate: -4.84; 95% CI: −6.07 - -3.61) were significantly associated with eGFR at follow-up. On the other hand, contrast agent volume showed no statistically significant effect (Estimate: 0.47; 95% CI: −0.52 - 1.46), even when adjusting for baseline kidney function and other clinical variables.

The results of the univariable and multivariable analyses regarding the association of KDIGO stage progression and eGFR at readmission are presented in Table 2. A separate graphical display can be found in Fig. 2, Fig. 3.

**Table 2:** Univariable and multivariable analyses of the association between contrast volume administered during PCI and risk of KDIGO stage progression (Cox proportional hazards model) and estimated glomerular filtration rate (linear regression analysis) at readmission.

	**Univariable**		**Multivariable**	
**KDIGO stage progression**	***p* value**	**HR (95% CI)**	**p value**	**HR (95% CI)**
**Contrast agent volume administered during index PCI**	0.1248	1.08 (0.98–1.19)	0.408	1.047 (0.94–1.17)
**Age**			**<0.001**	1.029 (1.02–1.04)
**Sex (female)**			0.646	1.03 (0.91–1.17)
**Body mass index (BMI)**			0.490	1.004 (0.99–1.02)
**Radial vascular access**			**0.006**	0.863 (0.78–0.96)
**KDIGO**				
G2			**<0.001**	0.232 (0.20–0.26)
G3a			**<0.001**	0.166 (0.14–0.20)
G3b			**<0.001**	0.073 (0.05–0.11)
G4			**<0.001**	0.114 (0.08–0.19)
**Diabetes mellitus**			**<0.001**	1.210 (1.09–1.34)
**Arterial hypertension**			**<0.001**	1.278 (1.14–1.44)
**Unstable angina at index PCI (vs. CCS)**			0.938	1.007 (0.84–1.21)
**NSTEMI at index PCI (vs. CCS)**			**<0.001**	1.571 (1.38–1.79)
**STEMI at index PCI (vs. CCS)**			**<0.001**	1.727 (1.48–2.01)
**eGFR at readmission**	**p value**	**Estimate (95% CI)**	**p value**	**Estimate (95% CI)**
**Contrast agent volume**	0.527	0.31 (−0.64–1.25)	0.352	0.47 (−0.52–1.46)
**eGFR pre-PCI**	**<0.001**	0.52 (0.50–0.54)	**<0.001**	0.47 (0.45–0.50)
**Age**			**<0.001**	-0.13 (−0.18 - -0.08)
**Sex (female)**			0.857	0.10 (−1.03–1.24)
**BMI**			0.630	0.03 (−0.08–0.13)
**Radial vascular access**			0.085	0.85 (−0.12–1.81)
**Diabetes mellitus**			**<0.001**	−3.13 (−4.11 - -2.14)
**Arterial hypertension**			**<0.001**	−2.88 (−3.90 - -1.86)
**Unstable angina at index PCI (vs. CCS)**			0.759	0.25 (−1.34–1.84)
**NSTEMI at index PCI (vs. CCS)**			**<0.001**	−4.84 (−6.07 - -3.61)
**STEMI at index PCI (vs. CCS)**			0.555	−0.46 (−1.98–1.06)

PCI denotes percutaneous coronary intervention; KDIGO denotes “Kidney Disease: Improving Global Outcomes” classification; CCS denotes chronic coronary syndrome; NSTEMI denotes Non-ST-Segment Elevation Myocardial Infarction; STEMI denotes ST-Segment Elevation Myocardial Infarction; eGFR denotes estimated glomerular filtration rate; BMI denotes Body mass index. Time to *Re*-PCI was included in the multivariable analysis and modeled with natural splines.

**Fig. 2:**
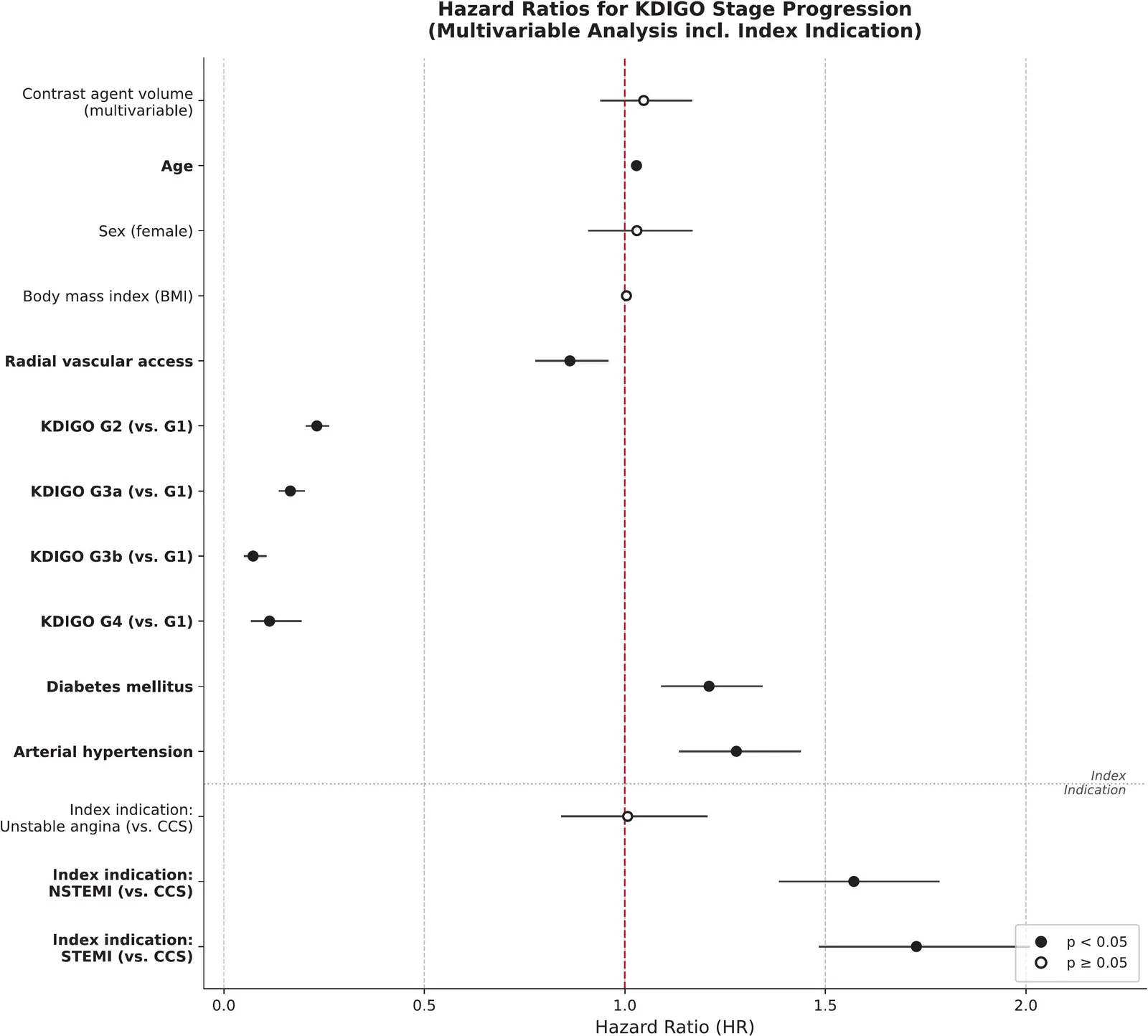
Multivariable analyses of the risk of KDIGO stage progression at readmission. BMI denotes body mass index; KDIGO denotes “Kidney Disease: Improving Global Outcomes” classification.; CCS denotes chronic coronary syndrome; NSTEMI denotes Non-ST-Segment Elevation Myocardial Infarction; STEMI denotes ST-Segment Elevation Myocardial Infarction.

**Fig. 3:**
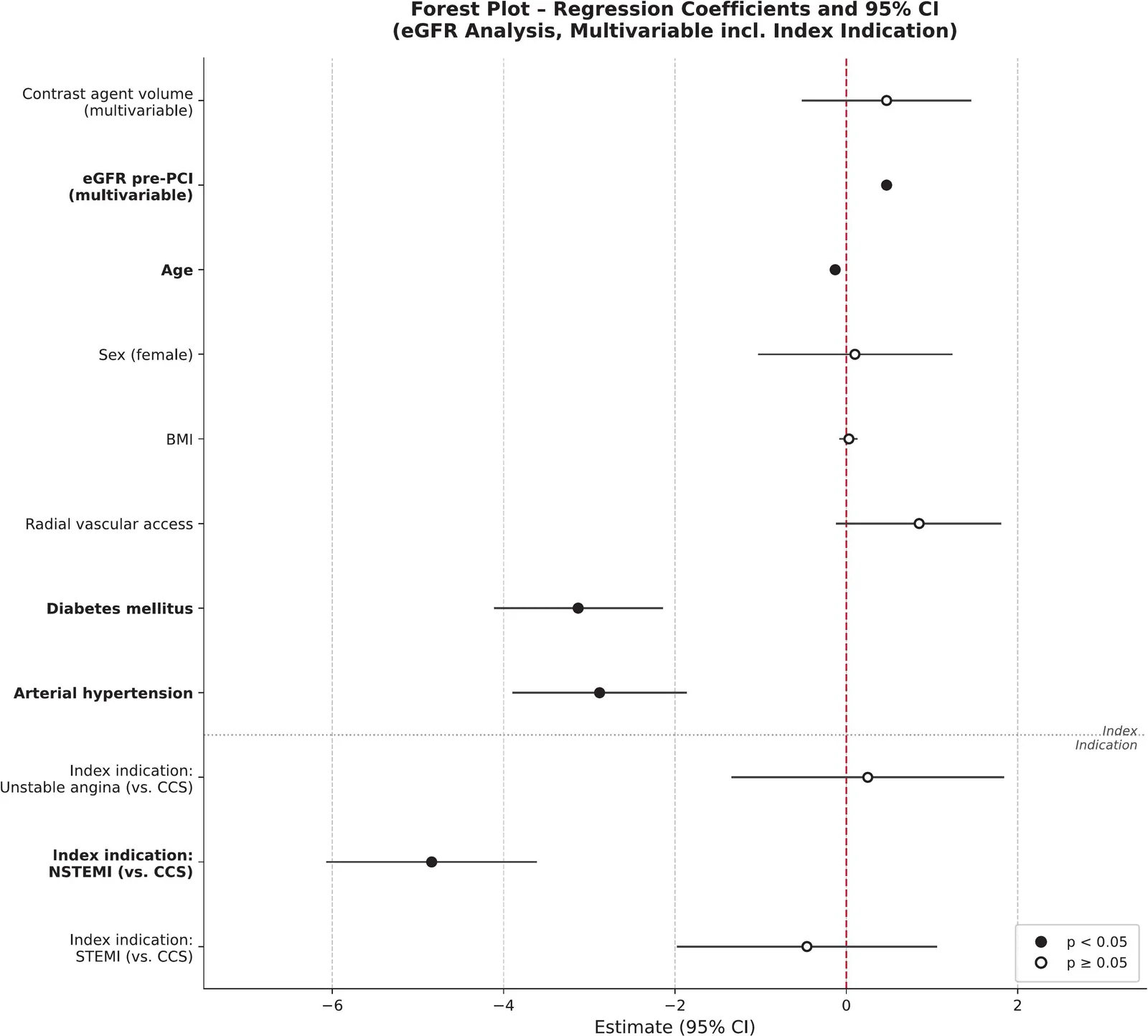
Multivariable analyses of the association between potential factors of renal outcomes and estimated glomerular filtration rate at readmission. PCI denotes percutaneous coronary intervention; eGFR denotes estimated glomerular filtration rate; BMI denotes Body mass index; CCS denotes chronic coronary syndrome; NSTEMI denotes Non-ST-Segment Elevation Myocardial Infarction; STEMI denotes ST-Segment Elevation Myocardial Infarction.

### 3.4 Sensitivity Analyses on the Impact of Contrast Agent Volumes Exceeding the Individual MACD on Medium- to Long-Term Renal Function

In 1814 of the patients (27%), the contrast agent volume administered during PCI exceeded the individual MACD. The median MACD was 300 mL [300,300]. The median volume of contrast administered in the group exceeding the MACD was substantially higher at 400 mL [330, 470] compared to 200 mL [150, 230] in the non-exceeding group.

At the time of the index PCI, the group exceeding the MACD had lower mean eGFR (68.5 vs. 76.6 mL/min/1.73 m^2^). Furthermore, a greater proportion of patients in this group were classified in more advanced KDIGO stages (G3a–G5: 31.8% vs. 19.1%), with the most pronounced difference observed in stage G5 (5.6% vs. <0.1%). Initial PCI in the MACD-exceeding group was more frequently performed via femoral access route (75.4% vs. 54.5% in the non-exceeding group).

Baseline characteristics, the time interval between procedures, the clinical presentation, and the prevalence of arterial hypertension and diabetes mellitus were similar between groups.

At the time of hospital readmission, the mean eGFR in the MACD-exceeding group was 69.25 mL/min/1.73 m^2^. The proportion of patients in KDIGO stages G3a to G5 was 30.3%. In the group that did not exceed the MACD, mean eGFR at follow-up was 73.24 mL/min/1.73 m^2^ (± 20.4), with 24.4% of patients falling into KDIGO stages G3a to G5.

A detailed summary is provided in Table 3.

**Table 3:** Baseline Characteristics Stratified by Contrast Agent Volume Above MACD Threshold.

	≤MACD (*n* = 4897)	>MACD (*n* = 1814)	Overall (*n* = 6711)
**MACD (mL)**	300 [300, 300]	300 [300, 300]	300 [300, 300]
**Administered contrast agent volume (mL)**	200 [150, 230]	400 [330, 470]	220 [170, 300]
**KDIGO at initial PCI**			
G1	1296 (26.5)	414 (22.8)	1710 (25.5)
G2	2658 (54.3)	822 (45.3)	3480 (51.9)
G3a	629 (12.8)	242 (13.3)	871 (13.0)
G3b	261 (5.3)	153 (8.4)	414 (6.2)
G4	51 (1.0)	82 (4.5)	133 (2.0)
G5	2 (0.0)	101 (5.6)	103 (1.5)
**KDIGO at re-PCI**			
G1	1104 (22.5)	376 (20.7)	1480 (22.1)
G2	2595 (53.0)	888 (49.0)	3483 (51.9)
G3a	711 (14.5)	252 (13.9)	963 (14.3)
G3b	364 (7.4)	155 (8.5)	519 (7.7)
G4	87 (1.8)	63 (3.5)	150 (2.2)
G5	36 (0.7)	80 (4.4)	116 (1.7)
**eGFR at initial PCI (mL/min/1.73m**^**2**^**)**	76.6 (± 19.0)	68.5 (± 26.1)	74.4 (± 21.5)
**eGFR at re-PCI (mL/**min**/1.73m**^**2**^**)**	73.2 (± 20.4)	69.3 (± 24.3)	72.2 (± 21.6)
**Age**	67.2 (± 10.4)	67.3 (± 10.5)	67.2 (± 10.4)
**Male**	3857 (78.8)	1527 (84.2)	5384 (80.2)
**BMI (kg/m**^**2**^**)**	27.9 (± 4.3)	28.2 (± 4.5)	28.0 (± 4.3)
**Vascular access route**			
Femoral	2671 (54.5)	1367 (75.4)	4038 (60.2)
Radial	2110 (43.1)	390 (21.5)	2500 (37.3)
**Interval between procedures (days)**	125 [66, 191]	132 [70, 191]	127 [67, 191]
**Diabetes mellitus**	1451 (29.6)	567 (31.3)	2018 (30.1)
**Arterial hypertension**	3574 (73.0)	1335 (73.6)	4909 (73.1)
**Clinical presentation**			
Acute coronary syndrome	1765 (36.0)	617 (34.0)	2382 (35.5)
STEMI	578 (11.8)	99 (5.5)	677 (10.1)
NSTEMI	757 (15.5)	337 (18.6)	1094 (16.3)
Unstable angina	430 (8.8)	181 (10.0)	611 (9.1)
Chronic coronary syndrome	3132 (64.0)	1197 (66.0)	4329 (64.5)

Sensitivity Analysis, values are mean ± SD or median [interquartile range] where appropriate, n (%). MACD denotes maximum allowable contrast dose; KDIGO denotes “Kidney Disease: Improving Global Outcomes” classification; PCI denotes percutaneous coronary intervention; eGFR denotes estimated glomerular filtration rate; BMI denotes Body mass index; STEMI denotes ST-Segment Elevation Myocardial Infarction; NSTEMI denotes Non-ST-Segment Elevation Myocardial Infarction.

### 3.5 Univariable and multivariable analyses of the Impact of Contrast Agent Volumes Exceeding the Individual MACD on a progression to a higher KDIGO stage and/or eGFR at readmission

In the univariable interval-censored Cox regression analysis, the administration of a contrast volume exceeding the individual MACD during PCI was not significantly associated with an increased risk of a transition to a higher KDIGO stage at follow-up (HR 0.93; 95% CI: 0.83–1.04).

In the multivariable analysis, exceeding the individual MACD during the index PCI again showed no statistically significant association with an increased risk of progression to a higher KDIGO stage at readmission (HR 1.00; 95% CI: 0.89–1.12). In contrast, older age, diabetes mellitus, arterial hypertension, not using a radial vascular access, STEMI and NSTEMI were significantly associated with a higher likelihood of progression to a worse KDIGO stage. Conversely, higher baseline KDIGO stages prior to PCI were significantly associated with a reduced risk of further deterioration.

In the univariable linear regression analysis, exceedance of the individual MACD during the initial PCI was not statistically significantly associated with eGFR levels at follow-up (Estimate: 0.22; 95% CI: −0.79-1.24).

In the corresponding multivariable analysis, exceeding the individual MACD during PCI remained not statistically significantly associated with eGFR levels at follow-up (Estimate: 0.27; 95% CI: −0.77–1.32). In contrast, diabetes mellitus, arterial hypertension, age, and NSTEMI were independently associated with eGFR at the time of hospital readmission.

A detailed overview of the results from the univariable and multivariable analyses is presented in Table 4.

**Table 4:** Univariable and multivariable analyses of associations between exceedance of the maximum allowable contrast dose and risk of KDIGO stage progression (Cox proportional hazards model) and estimated glomerular filtration rate (linear regression analysis) at readmission.

	Univariable		Multivariable	
KDIGO stage progression	p value	HR (95% CI)	p value	HR (95% CI)
**Exceedance of MACD during index-PCI**	0.194	0.93 (0.83–1.04)	0.996	1.00 (0.89–1.12)
**Age**			**<0.001**	1.03 (1.02–1.04)
**Sex (female)**			0.693	1.03 (0.90–1.16)
**BMI**			0.440	1.01 (0.99–1.02)
**Radial vascular access**			**0.003**	0.85 (0.77–0.95)
**KDIGO**				
G2			**<0.001**	0.23 (0.20–0.26)
G3a			**<0.001**	0.17 (0.14–0.20)
G3b			**<0.001**	0.07 (0.05–0.11)
G4			**<0.001**	0.11 (0.07–0.19)
**Diabetes mellitus**			**<0.001**	1.21 (1.09–1.34)
**Arterial hypertension**			**<0.001**	1.28 (1.13–1.44)
**Unstable angina at index PCI (vs. CCS)**			0.956	1.01 (0.84–1.20)
**NSTEMI at index PCI (vs. CCS)**			**<0.001**	1.57 (1.39–1.79)
**STEMI at index PCI (vs. CCS)**			**<0.001**	1.72 (1.48–2.00)
**eGFR at readmission**	**p value**	**Estimate (95% CI)**	**p value**	**Estimate (95% CI)**
**Exceedance of MACD during index-PCI**	0.666	0.22 (−0.79–1.24)	0.611	0.27 (−0.77–1.32)
**eGFR pre-PCI**	**<0.001**	0.52 (0.50–0.54)	**<0.001**	0.47 (0.45–0.50)
**Age**			**<0.001**	−0.13 (−0.18 - -0.08)
**Sex (female)**			0.872	0.09 (−1.04–1.22)
**BMI**			0.592	0.03 (−0.08–0.13)
**Radial vascular access**			0.060	0.91 (−0.04–1.85)
**Diabetes mellitus**			**<0.001**	−3.18 (−4.16 - -2.19)
**Arterial hypertension**			**<0.001**	−2.83 (−3.86 - -1.81)
**Unstable angina at index PCI (vs. CCS)**			0.776	0.23 (−1.36–1.82)
**NSTEMI at index PCI (vs. CCS)**			**<0.001**	−4.82 (−6.05 - -3.59)
**STEMI at index PCI (vs. CCS)**			0.571	−0.44 (−1.95–1.08)

MACD denotes maximum allowable contrast dose; KDIGO denotes “Kidney Disease: Improving Global Outcomes” classification; PCI denotes percutaneous coronary intervention; BMI denotes Body mass index; CCS denotes chronic coronary syndrome; NSTEMI denotes Non-ST-Segment Elevation Myocardial Infarction; STEMI denotes ST-Segment Elevation Myocardial Infarction; eGFR denotes estimated glomerular filtration rate. Time to *Re*-PCI was included in the multivariable analysis and modeled with natural splines.

## 4 Discussion

To the best of our knowledge, this large, real-world study is among the most comprehensive investigations of the impact of contrast volume during PCI on medium- to long-term renal function. While previous research has focused almost exclusively on acute renal effects within the first few days after contrast exposure, our study included a substantially longer follow-up (median 127 days). The key findings are:

1. Contrast volume during PCI was not associated with medium- to long-term deterioration of renal function.
2. This lack of association persisted even when investigating whether the contrast volume exceeded the patient-specific MACD.
3. Deterioration of medium- to long-term renal function was instead driven by pre-existing predispositions, comorbidities and clinical presentation.
4. The choice of arterial vascular access was significantly associated with renal function upon readmission with a lower risk of decline in renal function when using a radial access.

Previous studies have demonstrated that the use of large contrast volumes during cardiovascular interventions – for instance those exceeding the individual MACD - was associated with an increased risk of CA-AKI [1], [5], [17], [18], [19], [20]. Consequently, contrast volume has long been considered a key, modifiable risk factor in routine clinical practice. In our study, mean renal function at readmission was indeed slightly worse compared to the time of the initial PCI with about a quarter of the patients having worsened KDIGO stages. However, crucially, the contrast volume used during PCI showed no statistically significant association with medium- to long-term renal function, regardless of whether renal function was assessed as a change in KDIGO stage or directly via eGFR. It should be noted that the normalization of contrast volume - while statistically appropriate to account for inter-individual differences in renal reserve - may reduce informative value at higher absolute contrast volumes, as the relative metric may not fully capture the nephrotoxic burden in patients receiving large absolute amounts of contrast. The dedicated subgroup analysis of patients exceeding the individually calculated MACD was therefore included to complement the primary analysis and to address this potential limitation directly. Notably, even exceeding the MACD was however not linked to a decline in renal function, despite the fact that in this subgroup - characterized by an already reduced baseline renal function - the median amount of contrast agent administered was twice as high.

Instead, the renal impairments associated with contrast agent use observed in clinical practice appear to be not induced by the contrast media itself but primarily attributable to preexisting renal risk factors, most notably age, arterial hypertension, and diabetes mellitus, as well as to the clinical presentation at index PCI: both STEMI and NSTEMI were independently associated with a higher risk of KDIGO stage progression, an association most likely reflecting the hemodynamic compromise inherent to acute coronary syndromes rather than a direct nephrotoxic effect of contrast media. These findings are largely in line with recent data on acute renal effects of intravenously administered contrast agents [13], which in many non-cardiac fields has already prompted a paradigm shift in recent years [21], but has not previously been demonstrated in the setting of cardiovascular interventions. Our study, however, suggest that – despite pathophysiological considerations [13], [21] - there may be no relevant difference between intravenous and intraarterial, catheter-based contrast administration with respect to its impact on renal function.

The vascular access used during PCI is considered another modifiable risk factor for the development of CA-AKI [6–7|. Large randomized trials and meta-analyses have shown that femoral access, compared to radial access, is associated with a higher risk of CA-AKI [8], [9]. This is one of the reasons why transradial access is generally recommended whenever feasible [1]. In our cohort, however, the initial PCI was most commonly performed via femoral access. This is likely explained by the study's observation period, which begins in 2003 - a time when femoral access was still considered standard practice. Still, radial vascular access was independently associated with a lower risk of KDIGO stage progression at readmission in the multivariable analysis. This finding aligns with the established evidence from acute renal outcomes, further supporting the preferential use of transradial access whenever clinically feasible*.*

## 5 Conclusions

The volume of contrast agent administered during PCI showed no significant impact on medium- to long-term renal function in patients undergoing repeated procedures within a follow-up interval of ≥7 days to ≤12 months. Instead, renal outcomes after PCI appeared to be driven primarily by patient- and procedural-related factors rather than contrast exposure.

These findings suggest that current preventive strategies – particularly the routine strict minimization of contrast volume or reliance on predefined dose thresholds - may merit re-evaluation. An individualized approach could prevent the potential under-treatment of patients requiring essential cardiovascular interventions despite high contrast needs.

### 5.1 Limitations

This study has several limitations that should be acknowledged. Although it represents a real-world population, it is based on a retrospective design, which inherently carries limitations related to data completeness, unmeasured confounding, and selection bias.

Furthermore, the study cohort exclusively included patients who underwent a repeat PCI within the defined follow-up period. As such, there is no direct comparison to the majority of patients who do not require a second intervention after an initial PCI. This represents an important limitation, as the cohort is inherently selective and likely enriched for patients with more advanced coronary artery disease or greater overall comorbidity burden. Importantly, given the retrospective nature of the study, several potentially relevant unmeasured factors may have influenced the results of this study on renal outcomes. Accordingly, caution is warranted when extrapolating these findings to broader or lower-risk populations, and preferably prospective studies with standardized protocols will be needed to confirm these results.

Furthermore, while clinical presentation at the time of index PCI was incorporated into the multivariable models as an additional covariate, adjustment for clinical presentation at the repeated procedure was not performed for methodological reasons. The primary endpoint analysis employs an interval-censoring approach, in which a change in KDIGO stage is observed between the index PCI and the repeated procedure, but the precise timing of this change remains unknown. Adjusting for the clinical indication of the repeated procedure would therefore risk introducing reverse causation bias in cases where an acute presentation occurred subsequent to – rather than prior to - the rise in KDIGO stage. This temporal ambiguity precludes a valid adjustment for re-PCI indication within the current analytical framework.

Additionally, the normalization of contrast volume to patient-specific parameters, while methodologically sound, may inherently limit sensitivity at the upper end of the distribution. Thus, absolute contrast volumes were also examined in the MACD-exceedance subgroup to mitigate this concern, though residual loss of informative value at extreme values cannot be entirely excluded.

Furthermore, only the impact on renal function up to a maximum of 12 months after the initial PCI was analyzed. The effect of the initial contrast volume on renal function beyond this time frame remains unclear, although sudden and drastic changes associated with the initial contrast use beyond this period appear highly unlikely.

Finally, in patients who underwent multiple re-PCIs within the observation window, only the first re-PCI was included in the analysis. This approach was chosen to reduce potential confounding due to repeated contrast exposures. However, it also limits conclusions about the cumulative effect of multiple contrast administrations over time.

## Acknowledgement of grant support

There was no grant support related to this study.

## CRediT authorship contribution statement

**Philipp Steinhoff:** Writing – review & editing, Writing – original draft, Visualization, Validation, Resources, Project administration, Methodology, Investigation, Formal analysis, Data curation, Conceptualization. **Christian M. Valina:** Validation, Resources, Conceptualization. **Derek Hazard:** Formal analysis, Data curation. **Miroslaw Ferenc:** Resources, Conceptualization. **Malte Niehoff:** Data curation, Conceptualization. **Dirk Westermann:** Supervision, Software, Resources. **Franz-Josef Neumann:** Writing – review & editing, Validation, Supervision, Software, Resources. **Philipp Breitbart:** Writing – review & editing, Visualization, Validation, Supervision, Methodology, Investigation, Conceptualization. **Philipp Ruile:** Writing – review & editing, Visualization, Validation, Supervision, Project administration, Conceptualization.

## Ethics approval

The study was approved by the institutional review board of the University of Freiburg (ethics approval reference number 23–1214-S1-retro, dated 09.07.2024).

## Sources of funding

There was no funding involved in this study.

## Declaration of competing interest

The authors declare that they have no known competing financial interests or personal relationships that could have appeared to influence the work reported in this paper.
